# EPIDEMIOLOGICAL FEATURES OF URINATION AND DEFECATION DYSFUNCTION: INSIGHTS FROM THE CHARLS STUDY (2011–2020)

**DOI:** 10.1093/geroni/igae098.2777

**Published:** 2024-12-31

**Authors:** Haoran Zhu, Jing Zhang, Guangtian Liu, Liwei Jing, Rui Wang, Yan Ye

**Affiliations:** Capital Medical University, Beijing, Beijing, China (People’s Republic); Capital Medical University, Beijing, Beijing, China (People’s Republic); North China University of Science and Technology, Tangshan, Hebei, China (People’s Republic); Capital Medical University, Beijing, Beijing, China (People’s Republic); Capital Medical University, Beijing, Beijing, China (People’s Republic); Capital Medical University, Beijing, Beijing, China (People’s Republic)

## Abstract

With the population aging, China has over 42 million older adults with disabilities, experiencing Urination and Defecation Dysfunction (UDD). The study aims to understand the epidemiological characteristics of UDD from 2011 to 2020, utilizing the China Health and Retirement Longitudinal Study (CHARLS) database. CHARLS is a longitudinal study, involved adults aged 45 and older, with having a cross-sectional survey at each two years. All participants in the CHARLS database who had UDD were included in the study. Chi-square tests and a logistic regression analysis were conducted. A total of 17,156, 17,897, 17,715, 19,097 and 19,129 eligible participants were recruited in each wave of survey, respectively, while associated prevalence were 4.4%, 4.0%, 4.7%, 4.7%, and 5.0%. The prevalence remained stable without significant changes across the ten years. In the univariable analysis, a higher prevalence was observed among older adults over 76 years (P< 0.05), compared to younger adults. Urban residents had a higher prevalence in Northern, Central and Southern China (P< 0.05) than other regions. Furthermore, through the multivariable analysis with the logistic model, higher UDD prevalence was identified among urban older female in Northern, Central, Southern, and Southwest China. Therefore, health educational campaigns and medical intervention programs should be provided for these high-risk population in China. The findings emphasize the potential burden of UDD in older populations, and underscore the importance of integrating urinary and fecal incontinence into geriatric health education.

